# Testicular Germ Cell Tumor Presenting as Torsion in a Young Patient

**DOI:** 10.7759/cureus.27506

**Published:** 2022-07-31

**Authors:** Omran Hasan, Ahmed A Al Rashed, Qasim M Isa, Nader Awad

**Affiliations:** 1 Urology, Salmaniya Medical Complex, Manama, BHR

**Keywords:** scrotal mass, scrotal exploration, urological malignancy, acute scrotum, testicular torsion, testicular germ cell tumors

## Abstract

Testicular torsion is a relatively common urological emergency, which involves the twisting of the spermatic cord and its contents leading to ischemia to the testes, which usually presents as sudden, severe scrotal pain. In comparison, testicular neoplasms are far less commonly encountered in the emergency department as they often present as painless hard masses that grow slowly over longer periods of time. Extremely rare cases of testicular neoplasms present as sudden scrotal pain that causes a challenging task in the emergency department as physical examinations and ultrasound findings could vary and not be specific enough in confirming the diagnosis.

In this case, we report a 22-year-old male who was referred from the emergency department (ED) as a case of testicular torsion from the presenting history; however, his physical examination and Doppler ultrasound findings were suspicious of testicular malignancy. The patient presented with a history of right scrotal pain for a few hours with no predisposing factors; however, examination and imaging were highly suspicious of an underlying neoplasm. The patient underwent an inguinal orchidectomy, and histology confirmed the presence of a germ cell tumor of varying components.

In conclusion, a high index of suspicion for testicular torsion should always be present when a patient presents with sudden onset testicular pain; however, the differential diagnosis including testicular neoplasms should not be overlooked as it can change the management and outcome.

## Introduction

The annual incidence of testicular neoplasms is around two to three cases per 100,000 male population in the United States [1], out of which approximately 90%-95% are confirmed to be germ cell tumors [1]. The commonest presentation of a testicular neoplasm is a hard, painless, slow-growing mass felt in the scrotum [1]. Nonetheless, they have occasionally presented in other ways such as chronic dullness or ache, a change in testicle shape on palpation, or even a dull pain felt in the lower abdomen [2]. However, the current literature describing acute painful presentations of testicular neoplasms demonstrates a varying rate of incidence, between 0.01% and 10%, of all cases [2], which illustrates the scarcity of such a presentation among the overall incidence of testicular neoplasms in the general male population [2].

In contrast to testicular cancer, testicular torsion is a relatively common urological emergency with the incidence reaching one in every 4000 males younger than 25 years in the United States per annum [3]. It involves the twisting of the spermatic cord and its contents leading to testicular ischemia and possible necrosis [3]. These cases present with a sudden onset of severe scrotal pain, usually starting within a few hours prior to presentation [3]. Consequently, the differential diagnosis of an intra-scrotal testicular tumor can be easily overlooked owing to the fact that most urologists, due to the high index of suspicion, will put torsion at the top of the differential diagnosis in any young patients presenting with acute scrotal pain.

In this case, we report a 22-year-old male who was referred from the emergency department (ED) as a case of testicular torsion from the presenting history; however, his physical examination and Doppler ultrasound findings were suspicious of testicular malignancy.

## Case presentation

A medically fit 22-year-old male presented to ED with a history of right testicular pain for a duration of four hours. The patient reported that the pain had started acutely with no predisposing factors or trauma. Upon scrotal examination, the right hemiscrotum appeared to be swollen and erythematous with the right testis being extremely tender to touch. In addition to that, a hard mass was also palpable, and this is what initially raised the possibility of an underlying malignancy versus the initial impression of testicular torsion. Furthermore, his initial investigative workup including a full blood count and urine analysis was unremarkable.

**Figure 1:**
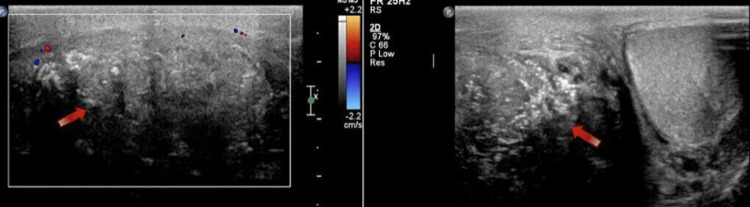
Color Doppler ultrasound showing disrupted, heterogenous right testis in the lateral view (left) and anterior view (right)

A color Doppler ultrasonography (CDUS) was arranged urgently and revealed a heterogenous appearance of the right testis with no evidence of normal testicular tissues as well as multiple macrocalcifications and cystic changes; however, no evidence of torsion of the right spermatic cord was noted (Figure 1).

Once these findings were observed, the diagnosis of a testicular neoplasm was highly suspected. Consequently, the condition was discussed in detail with the patient and his family, and they were informed of the possibility of an underlying testicular neoplasm. The patient was informed that he will require surgery for testicular exploration with a high possibility of right orchidectomy through an inguinal approach.

Moreover, prior to surgery, blood tumor markers were sent and showed an elevated alpha-fetoprotein (AFP) of 136.9 µg/L, beta-human chorionic gonadotropin (beta-HCG) of 942.1 mIU/mL, and lactate dehydrogenase (LDH) of 555 U/L adding to the diagnosis of an underlying testicular tumor. The patient was taken for right inguinal orchidectomy, and intraoperatively, the right testis appeared as a hard mass without twisting of the spermatic cord, hence confirming the absence of testicular torsion. The spermatic cord was ligated near the internal inguinal ring, and an inguinal orchidectomy was performed with the right testis sent for histopathology review.

**Figure 2:**
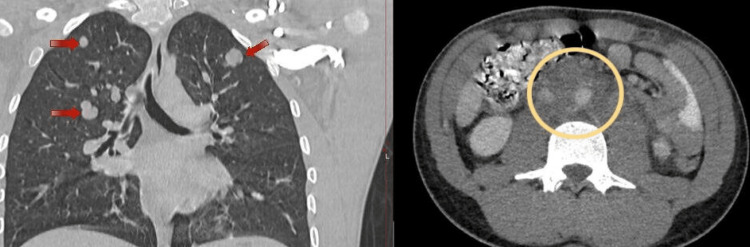
CT scan of the abdomen and chest for cancer staging demonstrating bilateral pulmonary metastasis (left - red arrow) and multiple retroperitoneal lymph nodal involvement (right - encircled in yellow) CT scan of the abdomen and chest for cancer staging demonstrating bilateral pulmonary metastasis (left - red arrow) and multiple retroperitoneal lymph nodal involvement (right - encircled in yellow)

The patient was discharged on postoperative day 2 in a stable condition. The right testicle histology report exhibited a mixed germ cell tumor with components described as 50% embryonal carcinoma, 35% teratoma, and 15% yolk sac tumor with lymphovascular invasion. Following histological confirmation of the diagnosis, a staging computerized tomography (CT) scan of the chest, abdomen, and pelvis was done and denoted pulmonary metastasis with extensive involvement of the retroperitoneal and common iliac lymph nodes (Figure 2).

Moreover, there was no evidence of bone or liver metastasis. Given this information, the staging of this patient’s neoplasm was regarded as T2N2M1. Finally, the case was presented in the National Tumor Board multidisciplinary team meeting and referred to the medical oncology team for further management.

## Discussion

Acute testicular pain in all prepubertal and young adult males should be considered as testicular torsion until proven otherwise. This makes prompt diagnosis and management to be of paramount importance since earlier time to surgical intervention is directly proportional to future testicular viability [3]. In comparison to the common incidence of torsion per annum, testicular germ cell tumors are relatively rare accounting for only 1%-2% of cancers among men in the United States [4].

The recognized presentation of testicular neoplasms is usually contradictory to that of testicular torsion, in which the earlier presents as a slow-growing painless mass over months and the latter as a sudden severe scrotal pain over hours [4] adding to the difficulty in diagnosing neoplasms, which present with sudden scrotal pain. An example of a case in which a tumor presented with acute pain leading to a misdiagnosis was reported by Alrabeeah et al. in 2017 [5] who described a case of testicular seminoma presenting as an acute scrotum [5]. He described the causes that led to the masking of the diagnosis, in that case, were the inability to properly assess the testis on physical examination, caused by the surrounding scrotal edema, as well as the nonspecific changes on ultrasound imaging, which led to a provisional diagnosis of epididymo-orchitis. This ultimately led to a delay in the ideal management [5]. That being said, the causes of sudden scrotal pain in testicular cancer patients have been previously described in the literature and were mostly attributed to the tumor itself. It was noted that in such cases, there was evidence of intratesticular bleeding within the tumor itself or even torsion due to its mass effect of the tumor, both causing testicular cancer to present as acute scrotum [6].

In addition to that, it has been reported that color Doppler ultrasound imaging has a high sensitivity 100% [7] and specificity of 97.9% [7] and is an invaluable tool aiding in the diagnosis of testicular torsion versus other pathologies. However, in the rare case of a testicular neoplasm presenting with acute pain, the diagnosis can be obscured by either severe scrotal edema or even concurrent torsion caused by the tumor itself [7]. Hence, the high index of suspicion for testicular torsion should always be the priority when a patient presents with sudden onset testicular pain, but testicular neoplasms should never be overlooked within the differential diagnosis. This is significant as it alters both the surgical approach as well as the patient's right to be informed that an orchidectomy will be have to be performed in contrast to an isolated testicular torsion case in which the main goal is to perform an orchidopexy and salvage the torted testis.

## Conclusions

In conclusion, as illustrated in this case, proper physical examination and CDUS aided in early recognition of the underlying testicular neoplasm rather than testicular torsion, and this prevented immediate scrotal exploration through a scrotal approach; however, as seen across the sparse literature regarding similar cases, the diagnosis can be easily missed.
